# The role of FoxO3a in the pathogenesis of osteoarthritis and its therapeutic applications

**DOI:** 10.3389/fimmu.2025.1650194

**Published:** 2025-09-23

**Authors:** Zhimin Wu, Xiaofei Wang, Yuxia Yang, Cunyi Xia, Linbing Lou, Wenyong Fei, Jingcheng Wang, Jihang Dai

**Affiliations:** ^1^ The Yangzhou School of Clinical Medicine of Dalian Medical University, Dalian, China; ^2^ Department of Orthopedics, Northern Jiangsu People’s Hospital Affiliated to Yangzhou University, Yangzhou, China; ^3^ Department of Orthopedics, Shandong Provincial Hospital Affiliated to Shandong First Medical University, Jinan, China

**Keywords:** FoxO3a, osteoarthritis, chondrocytes, oxidative stress, transcription factor

## Abstract

Osteoarthritis (OA) is a chronic degenerative joint disease predominantly observed in middle-aged and elderly individuals, with its complex pathological mechanisms significantly affecting patients’ quality of life. Due to the absence of effective treatment strategies, there has been a growing emphasis on molecular targeted therapies for OA. As a critical transcription factor, Forkhead box O3a (FoxO3a) plays a vital role in physiological processes such as cell differentiation, survival, and apoptosis. The activity of FoxO3a is modulated by post-translational modifications, including phosphorylation and acetylation, as well as by various signaling pathways. Recent studies have demonstrated that FoxO3a significantly influences the onset and progression of OA by regulating multiple processes in chondrocytes, including redox homeostasis, inflammatory response, cell survival, and matrix degradation. Its active expression presents potential value for the prevention and treatment of OA. This article reviews the research advancements regarding the role of FoxO3a in the pathogenesis of OA, emphasizing its effects on physiological activities such as oxidative stress and regulatory mechanisms in chondrocytes, with the aim of refining the understanding of OA pathogenesis and providing new insights for its prevention and treatment.

## 1 Introduction

Osteoarthritis (OA) is a chronic degenerative joint disease characterized by the progressive degeneration of articular cartilage, subchondral bone remodeling, and synovitis (1). The primary clinical manifestations of OA include progressive joint pain, dysfunction, and joint deformation, which severely impact patients’ quality of life (2, 3). Under the combined influence of an aging population and rising obesity rates, the incidence of OA has shown a significant upward trend. Epidemiological data indicate that since 1990, the global prevalence of OA has increased by 132% (4). It is estimated that by 2021, the global number of OA patients reached 595 million, accounting for 7.6% of the global population. OA has become the leading joint disease causing disability in middle-aged and elderly individuals, resulting in a substantial socioeconomic burden. Notably, the economic burden of knee OA accounts for approximately 1.0-2.5% of the GDP in developed countries (5). Therefore, in-depth research into the pathogenesis and more effective therapeutic targets for OA has become the focus of current investigations.

OA is a complex and heterogeneous disease that involves the entire joint tissue, with lesions affecting the articular cartilage, synovium, ligaments, meniscus, and subchondral bone. Its pathogenesis involves the interplay of various factors, including mechanical stress, genetics, metabolism, inflammation, and oxidative stress (6). Under the pathological conditions of OA, chondrocytes undergo a series of changes, including phenotypic alterations, increased apoptosis, autophagy imbalance, the release of inflammatory factors, and oxidative stress damage, ultimately leading to cartilage matrix degradation and structural destruction of the joint (6, 7). Additionally, subchondral bone remodeling and synovitis also drive the progression of OA through different mechanisms (8, 9).

Transcription factors are a class of essential proteins that specifically bind to the regulatory sequences of target gene DNA, there by regulating their gene expression. In tumors, MYC interacts with various proteins through promoter binding, epigenetic modifications, and processes such as initiation, elongation, and post-transcriptional regulation, thereby driving cancer progression (10). In the hematopoietic system, transcription factors, including the Pu.1 and Gata family, determine the differentiation of stem cells into various blood cell lineages through combinatorial actions (11). Recently, the regulation of transcription factors in the pathogenesis of OA has emerged as a prominent research focus (12). Runt-related transcription factor 3 (RUNX3) protects articular cartilage by upregulating lubricin and aggrecan (ACAN). RUNX2 exhibits a bidirectional effect under inflammatory conditions by regulating the expression of type II collagen (COL2A1) and matrix metallopeptidase 13 (MMP13); its heterozygous deletion inhibits OA, while complete deletion accelerates cartilage degeneration (13). Deciphering the spatiotemporally specific and environment -dependent regulatory network formed by transcription factors in OA is crucial for elucidating the molecular mechanisms underlying OA condition.

FoxO3a is a member of the Forkhead box O (FoxO) transcription factor family. The mammalian FoxO family comprises of several transcription factors, including FoxO1, FoxO3, FoxO4, and FoxO6, all of which contain a relatively conserved forkhead DNA-binding domain (14). These transcription factors are involved in various physiological functions such as cell metabolism, redox homeostasis, proliferation, DNA repair, and autophagy by regulating the expression of target genes (15–17). Mice with a cartilage-specific knockout of FOXO1/3/4 (Col2Cre-TKO) exhibit cartilage degeneration, synovial thickening, and osteophyte formation by 4–6 months of age, accompanied by reduced expression of autophagy-related genes (18). The FoxO3a gene, located on chromosome 6q21, serves as a critical regulator of various physiological activities *in vivo*. It is significantly involved in the occurrence and progression of diseases, including diabetic cardiomyopathy, intervertebral disc degeneration, and breast cancer, through the regulation of cellular redox, suppression of tumor activity, and amelioration of inflammatory responses (19, 20). Its activity is primarily regulated by post-translational modifications (PTMs) such as phosphorylation, acetylation, and ubiquitination. For instance, protein kinase B (AKT)-mediated phosphorylation of FoxO3a leads to its retention in the cytoplasm and a loss of transcriptional activity, whereas the deacetylase silent information regulator 2 homolog 1 (SIRT1) activates FoxO3a, enhancing its stress resistance capabilities (21). Given the pivotal role FoxO3a plays in regulating cellular metabolism and survival, its involvement in OA has garnered increasing attention.

Considering the significant role of processes such as the imbalance of redox homeostasis in chondrocytes in the pathogenesis of OA, it is particularly important to delve into the specific role and regulation of FoxO3a in the development of OA (22, 23). This article comprehensively reviews the mechanisms of FoxO3a in OA and its therapeutic applications, focusing on aspects including structure, expression, function, regulation, and future prospects. Unlike other studies on similar topics, this review places a greater emphasis on exploring therapeutic strategies targeting FoxO3a and outlining future research directions, aiming to offer new insights into the pathogenesis and treatment strategies for OA.

## 2 Structure of FoxO3a

**Figure 1:**
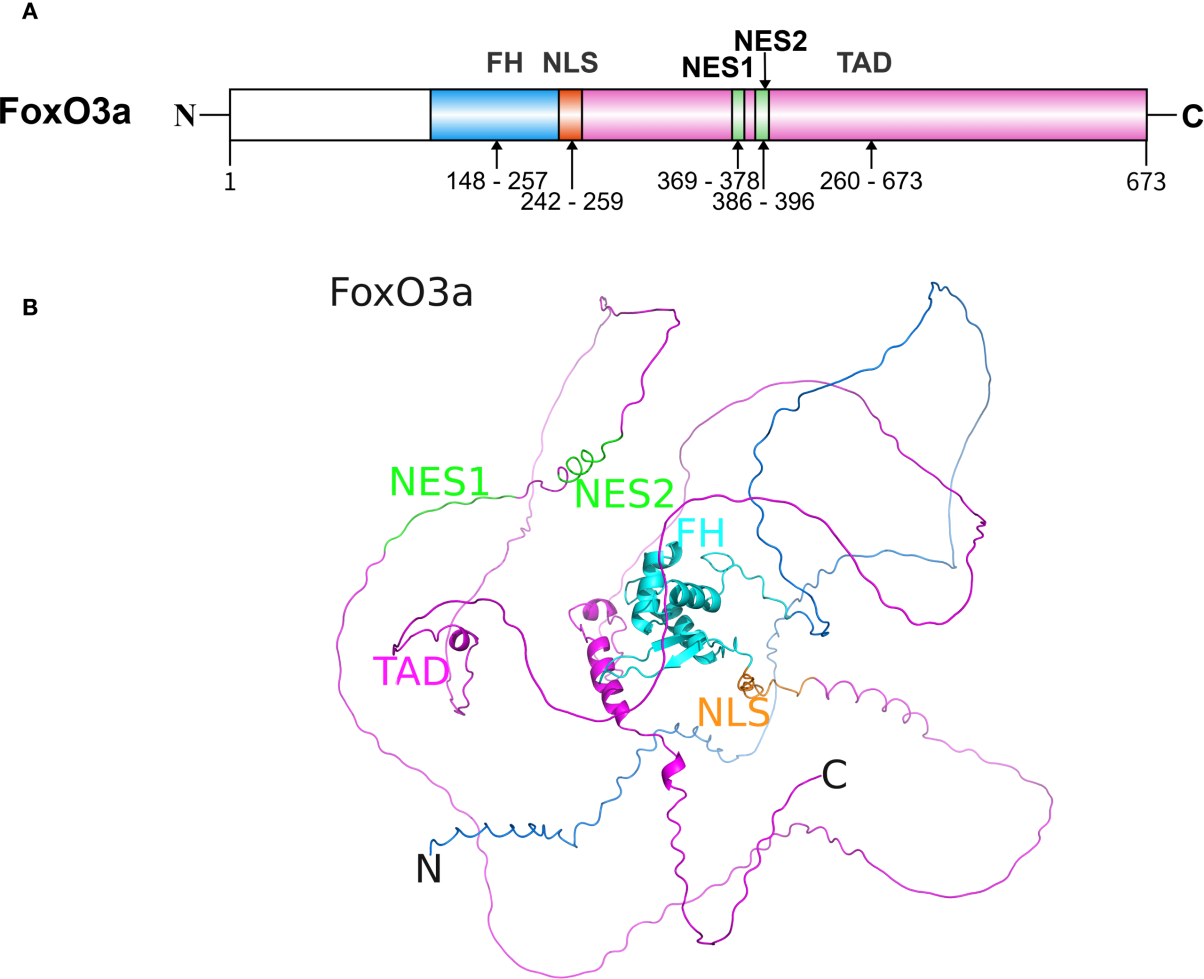
The structure of FoxO3a protein. **(A)** The sequence and functional domains of FoxO3a. The primary sequence of FoxO3a in humans consists of approximately 673 amino acid residues. **(B)** The predicted three-dimensional structure of FoxO3a (Created with AlphaFold 3). The well-conserved FH is flanked by disordered N- and C-termini. FH, forkhead DNA binding domain; NLS, nuclear localization sequence; NES, nuclear export sequence; TAD, transactivation domain.

FoxO3a belongs to the FoxO subfamily of transcription factors and consists of a forkhead (FH) domain, a nuclear localization sequence (NLS), two nuclear export sequences (NES), and a transactivation domain (TAD) (**Figure 1**) (24). The FH domain, located centrally within the protein, is a highly conserved DNA-binding domain comprising approximately 100 amino acids. This domain can recognize and bind to specific DNA sequences within the promoter regions of target genes, including manganese superoxide dismutase (MnSOD) and catalase (CAT), thereby regulating the transcription of these genes (25). The NLS and NES are responsible for regulating the subcellular localization of FoxO3a, determining its ability to enter the nucleus and initiate the transcription of target genes (26). The TAD contains multiple phosphorylation and other PTM sites. These PTMs, including phosphorylation, acetylation, ubiquitination, and methylation, are crucial mechanisms that regulate FoxO3a’s subcellular localization, DNA-binding ability, transcriptional activity, and stability, enabling it to integrate signals from various signaling pathways.

## 3 Expression of FoxO3a in articular cartilage and chondrocytes

FoxO3a is widely expressed across various human tissues and organs, participating in physiological processes such as cell proliferation, apoptosis, autophagy, and redox reactions. As a transcription factor, the active state of FoxO3a is primarily determined by its modification status and subcellular localization. When FoxO3a is localized within the nucleus and remains unmodified, it exhibits its transcription factor activity; conversely, when modified by processes such as phosphorylation, it is sequestered in the cytoplasm and rendered inactive.

Numerous studies have shown that under OA pathological conditions, the expression level, phosphorylation status, and subcellular localization of FoxO3a in chondrocytes may change, thereby influencing the phenotype and function of chondrocytes. Age is the principal risk factor for OA (27). In both human and murine articular cartilage, the expression of FoxO3a significantly declines with age, particularly in the superficial layer of weight-bearing regions, where the reduction in nuclear localization may correlate with diminished oxidative stress defense mechanisms (28, 29). Similarly, the expression of FoxO3a is notably reduced in knee cartilage samples from aged and OA mice (30).

In addition to aging, FoxO3a exhibits varying degrees of expression suppression in multiple OA models, both *in vivo* and *in vitro*. Its expression is significantly diminished in human OA cartilage and chondrocytes (31). In mouse chondrocytes, interleukin-1β (IL-1β) and tumor necrosis factor-α (TNF-α) induce phosphorylation and nucleocytoplasmic shuttling of FoxO3a, leading to a decreased expression level in the nucleus (32).

However, conflicting perspectives have emerged. Research indicates that in an IL-1β-treated rabbit chondrocyte inflammation model, FoxO3a is significantly upregulated and influences chondrocyte survival by modulating the expression of the oxidative stress marker inducible nitric oxide synthase (iNOS) and cellular apoptosis (33). Nevertheless, it is widely accepted that the dysregulation of FoxO3a activity is closely associated with the progression of OA.

## 4 Biological functions of FoxO3a in chondrocytes

### 4.1 Redox homeostasis

Aging induces chondrocytes to secrete a senescence-associated secretory phenotype (SASP), leading to mitochondrial dysfunction and imbalanced regulation of cell death through multiple mechanisms, including oxidative stress, inflammatory response, apoptosis, and autophagy, thereby promoting the development of OA (34).

Redox homeostasis is a dynamic balance of oxidation and reduction reactions within cells, maintained through various antioxidant enzymes, thioredoxin systems, and small molecule antioxidants (35–37). Oxidative stress refers to the imbalance between the production of reactive oxygen species (ROS), nitric oxide (NO), iNOS, and superoxides, and the antioxidant defense system. Excessive ROS can damage DNA, proteins, and lipids, induce chondrocyte apoptosis and senescence, provoke inflammatory responses, and promote cartilage matrix degradation (38). Antioxidant enzymes include SOD, CAT, and glutathione peroxidase (GPx). Notably, oxidative stress is not purely detrimental; moderate levels of ROS can function as signaling molecules that activate protective responses, exemplifying the ‘hormesis’ effect in homeostatic regulation (37, 39).

When chondrocytes are subjected to oxidative stress, FoxO3a is typically activated and translocated into the nucleus, leading to the upregulation of a series of antioxidant enzymes, including SOD, CAT, and GPx, which are crucial for eliminating intracellular ROS and mitigating oxidative damage to cells (40, 41). AMPK (5’ adenosine monophosphate-activated protein kinase) is a protein kinase found in eukaryotic organisms. Under oxidative stress conditions, AMPK establishes a multi-layered network for DNA repair and protection by directly regulating repair enzymes, activating SIRT3 to safeguard mitochondrial DNA, coordinating energy metabolism and autophagy, and interacting with DNA damage response factors (42, 43). AMPK activates FoxO3a in chondrocytes, resulting in the downregulation of various oxidative stress and catabolic markers, which alleviates mitochondrial oxidative damage and promotes DNA repair (30). FoxO3a-NETT@SMs have been shown to restore mitochondrial function and inhibit apoptosis in H_2_O_2_-induced OA chondrocytes through the overexpression of FoxO3a (31). Furthermore, DL-3-n-Butylphthalide (NBP) enhances FoxO3a expression by inhibiting the phosphatidylinositol 3-kinase (PI3K)/AKT pathway, which increases the expression of MnSOD and CAT, thus improving the chondrocytes’ ability to withstand oxidative stress and alleviating oxidative damage-induced apoptosis and matrix degradation (44). In contrast, knocking down FoxO1 and FoxO3a using tBHP resulted in a significant reduction in GPx-1 and CAT levels, exacerbating oxidative damage in chondrocytes (29). However, a study by Wang et al. indicated that silencing FoxO3a could reduce the upregulation of iNOS induced by IL-1β in rabbit chondrocytes, thereby providing protection against oxidative stress (**Table 1**) (33). Nevertheless, maintaining or enhancing the antioxidant function of FoxO3a remains a promising strategy for protecting chondrocytes from oxidative stress and delaying the progression of OA.

**Table 1:** Biological functions of FoxO3a in chondrocytes.

Biological process	Models	Mechanisms	Biological functions	Ref.
Redox Homeostasis	Human chondrocytes	FoxO3a activation leads to the upregulation of SOD2 and the inhibition of NO	Alleviates mitochondrial oxidative damage	(30)
	Human OA chondrocytes	Overexpression of FoxO3a mitigates increases in ROS	Alleviates mitochondrial oxidative damage	(31)
	Human OA chondrocytes and cartilage explants	Upregulation of FoxO3a enhances the expression of MnSOD	Inhibits oxidative stress	(44)
	Human chondrocytes	Silencing FoxO3a results in ROS accumulation	Inhibits oxidative stress	(29)
	IL-1β-treated rabbit chondrocytes	Silencing Foxo3a diminishes iNOS expression	Promotes oxidative stress	(33)
ECM Metabolism	ATDC5 cells	Silencing FoxO3a reduces the expression of Sox9	Promotes anabolic metabolism	(48)
	Human OA chondrocytes and DMM rats	Overexpression of FoxO3a increases the expression of COL2A1	Strengthens anabolic metabolism	(31)
	Human OA chondrocytes	Foxo3a activation downregulates MMPs	Inhibits catabolic metabolism	(44)
	Col2Cre-TKO mouse cartilage	Combined knockout of FoxO1/3a/4 inhibits Prg4 expression	Promotes anabolic metabolism	(18)
Apoptosis	IL-1β-treated ATDC5 cells	circFoxO3a enhances the expression of caspases	Reduces apoptosis	(53)
	Human chondrocytes	siFoxO3a induces the upregulation of caspases	Reduces apoptosis	(29)
	IL-1β or TNF-α-treated mouse chondrocytes	Activation of FoxO3a reduces the TUNEL positive rate	Improves apoptosis outcomes	(32)
	SCP4 knockout mouse cartilage and chondrocytes	Dephosphorylation of FoxO3a inhibit the expression of caspases	Reduces apoptosis	(54)
	IL-1β-treated rabbit chondrocytes	FoxO3a silencing inhibits apoptosis	Promotes apoptosis	(33)
	Human OA chondrocytes	FoxO3a upregulation inhibits the expression of Bax	Reduces apoptosis	(44)
Autophagy	Mouse chondrocytes	FoxO3a activation promotes the expression of Beclin-1	Enhances mitochondrial autophagy	(61)
	Human chondrocytes	FoxO3a activation leads to increased LC3-II	Activates autophagy	(62)
	Human chondrocytes and obese OA mouse cartilage	FoxO3a activation enhance Gabarapl1	Enhances autophagic flux	(63)
Differentiation	ATDC5 cells and mouse MSCs	Overexpression of FoxO3a induces the expression of ACAN	Promotes early chondrogenesis and terminal hypertrophic differentiation	(48)
	Human MSCs	Loss of FoxO3a leads to upregulation of ACAN	Inhibits hypertrophic differentiation	(64)
	OA rat cartilage	FoxO3a activation inhibits the expression of COL X	Inhibits OA cartilage hypertrophy	(65)
Senescence	DMM and aged mouse cartilage	FoxO3a upregulation inhibits the levels of P16	Antagonizes cartilage degeneration and senescence	(66)
	IL-1β-treated rat chondrocytes	FoxO3a deacetylation inhibits the levels of P21	Inhibits chondrocyte senescence	(67)

### 4.2 The synthesis and degradation of extracellular matrix

The extracellular matrix (ECM) is primarily composed of macromolecules, including collagen, glycoproteins, and ACAN, and its synthesis and degradation are regulated by various cytokines and growth factors (45). The function of articular cartilage is dependent on the integrity of the cartilage ECM. Chondrocytes play a crucial role in maintaining cartilage homeostasis by balancing the expression of matrix synthesis-related genes (e.g., ACAN, COL2A1, and Sox9) and matrix degradation-related genes including MMPs and a disintegrin and metalloproteinase with thrombospondin motifs (ADAMTSs) (46, 47).

The homeostasis of the cartilage ECM is closely associated with the activity of FoxO3a. In ATDC5 cells and human OA chondrocytes, overexpression of FoxO3a significantly increased the expression of Sox9, ACAN, and COL2A1, while its downregulation inhibited the expression of these markers (31, 48). Furthermore, NBP reduced the expression of ADAMTSs and MMPs in human OA chondrocytes by activating FoxO3a (44). In mouse models, cartilage-specific knockout of FoxO1/3a/4 resulted in spontaneous cartilage degradation and exacerbated OA lesions (**Table 1**) (18). In summary, the regulation of cartilage ECM homeostasis by FoxO3a contributes to the maintenance the integrity of articular cartilage integrity, thereby mitigating OA progression.

### 4.3 Apoptosis

Apoptosis, also known as programmed cell death, is a highly ordered physiological mechanism, characterized by distinctive morphological changes, including cell membrane shrinkage, chromatin condensation, and DNA fragmentation (49, 50). Chondrocyte apoptosis is a significant feature of cartilage degeneration in OA, leading to a reduction in both the number and activity of chondrocytes, as well as a decline in ECM synthesis capacity (51).

In various cell types, the accumulation of activated FoxO3a in the nucleus can upregulate the expression of pro-apoptotic genes, thereby inducing cell apoptosis (52). By inhibiting the active expression of FoxO3a, circFoxO3a enhances the expression of apoptosis markers, including Cleaved PARP, Cleaved caspase-3, and BAX, which are induced by IL-1β in ATDC5 cells, thus exacerbating cell apoptosis (53). Additionally, siFoxO3a can also induce chondrocyte apoptosis, accompanied by caspase activation (29). Punicalin ameliorates IL-1β and TNF-α-induced chondrocyte growth inhibition and apoptosis by preserving the transcriptional activity of FoxO3a (32). The protein phosphatase SCP4 promotes the nuclear translocation of FoxO3a through its dephosphorylation, thereby reducing chondrocyte apoptosis and promoting cartilage development (54). However, a study has indicated that FoxO3a, under IL-1β stimulation, exacerbates inflammatory responses by positively regulating tenascin-c (Tnc), promoting iNOS expression, and inducing apoptosis in rabbit chondrocytes (33). In summary, FoxO3a exerts anti-apoptotic effects on chondrocytes through antioxidation or inhibition of hypertrophic differentiation, especially when homeostasis or specific protective regulations are present (**Table 1**) (44).

### 4.4 Autophagy

Autophagy is an evolutionarily conserved intracellular degradation mechanism that recycles damaged proteins and organelles through the lysosomal pathway. This process involves autophagosome formation, substrate encapsulation, and fusion with lysosomes, thereby maintaining cellular homeostasis (55–57). In articular cartilage, moderate autophagy is crucial for maintaining chondrocyte function and matrix balance (58, 59).

Activated FoxO3a can enter the nucleus and upregulate the expression of various autophagy-related genes (Atgs), such as LC3, Beclin-1, Gabarapl1, and ULK1, thereby initiating or enhancing autophagic flux (60). The synergistic effect of FoxO3a and SIRT3 alleviates oxidative stress in mouse endplate chondrocytes by enhancing mitophagy and inhibiting NLRP3 inflammasome activation (61). Glucosamine activates autophagy in human chondrocytes by inhibiting the AKT/FoxO3a/mTOR pathway (62). Furthermore, adenosine A2A receptors enhance the autophagic flux of Gabarapl1 and Beclin-1 by activating FoxO1/3a, which improves cartilage metabolism and reduces apoptosis—a mechanism validated in obese OA mouse models (**Table 1**) (63). Therefore, maintaining appropriate FoxO3a activity may promote chondrocyte autophagy and delay the progression of OA.

### 4.5 Other biological functions

In addition to the aforementioned functions, FoxO3a also participates in regulating processes such as differentiation and senescence in chondrocytes. Overexpression of FoxO3a can induce the expression of various chondrocyte differentiation markers, including Sox9, ACAN, and COL2A1, thereby enhancing early chondrogenesis and terminal hypertrophy of cartilage stem cells (48). This suggests that FoxO3a may be involved in the differentiation of mesenchymal stem cells (MSCs) into chondrocytes. However, some studies have indicated that upregulation of FoxO3a can inhibit the hypertrophic differentiation of MSCs by reducing the expression of type X collagen (COL X), while also limiting excessive proliferation by promoting early apoptosis (64). The natural flavonoid 5,7,3’,4’-tetramethoxyflavone (TMF) effectively inhibits OA cartilage hypertrophy by activating FoxO3a (65). Furthermore, fibroblast growth factor 18 (FGF18) can inhibit the expression of P16, P21, and P53 by activating FoxO3a, thus protecting chondrocytes from degeneration and aging effects (66). Ubiquitin-specific protease 3 (USP3) upregulates SIRT3 to deacetylate FoxO3a and attenuates IL-1β-induced senescence in SD rat chondrocytes (**Table 1**) (67). These studies further expand our understanding of the complex roles of FoxO3a in OA.

## 5 Regulation of FoxO3a in chondrocytes

### 5.1 Upstream signaling pathways

The PI3K/AKT signaling pathway serves as a classic negative regulator of FoxO3a. Upon activation of the PI3K/AKT pathway by growth factors, AKT phosphorylates conserved amino acid sites on FoxO3a. Phosphorylated FoxO3a binds to 14-3–3 proteins and remains in the cytoplasm, inhibiting its entry into the nucleus and thereby preventing its transcriptional regulatory functions (21). In chondrocytes, AKT mediates the phosphorylation of FoxO3a at specific sites (e.g., Ser253), leading to its retention in the cytoplasm and subsequent inactivation, which affects the antioxidant and autophagy functions of chondrocytes (44, 68, 69).

The AMPK/SIRT pathway has the capacity to activate FoxO3a. Deacetylases, such as SIRT1, activate FoxO3a through deacetylation modifications, which enhance its DNA-binding ability and transcriptional activity, thereby promoting cellular resistance to oxidative stress and extending lifespan (70). TMF can facilitate the deacetylation of FoxO3a by activating SIRT1 in chondrocytes, which enhances its nuclear translocation and transcriptional activity (71). Similarly, USP3 promotes the deacetylation of FoxO3a by upregulating SIRT3, thus inhibiting IL-1β mediated senescence in rat chondrocytes (**Table 2**) (67).

**Table 2:** Regulatory mechanisms of FoxO3a in chondrocytes.

Regulation type	Regulation methods	Mechanisms	Biological functions	Expression of FoxO3a	Ref.
Signaling Pathways	PI3K/AKT	AKT promotes FoxO3a phosphorylation and keeps it in the cytoplasm	Reduces antioxidant and autophagy functions	↓	(44, 68, 69)
	AMPK/SIRT1	SIRT1 Induces FoxO3a deacetylation and its nuclear translocation	Boosts antioxidant and autophagy functions	↑	(71)
	USP3/SIRT3	SIRT3 induces FoxO3a deacetylation	Inhibits cellular senescence	↑	(67)
PTMs	Phosphorylation	AMPKα induces FoxO3a phosphorylation	Promotes expression of MMPs	↓	(73)
	Deacetylation	SIRT1 removes acetyl groups from FoxO3a	Activates antioxidant functions	↑	(67, 71)
Epigenetics	miRNAs	miR-182 targets 3’UTR of FoxO3a	Promotes inflammatory response	↓	(77)

↑/↓ indicates that the expression of FoxO3a is up/down-regulated.

### 5.2 Post-translational modifications

As a transcription factor, FoxO3a’s intracellular localization is heavily dependent on its amino acid modifications. The removal of these modifications allows FoxO3a to enter the nucleus, where it binds to the promoter regions of target genes and regulates transcription (72). Phosphorylation and acetylation are the most extensively studied post-translational modifications (PTMs) of FoxO3a. Besides AKT, other kinases can also phosphorylate FoxO3a. For instance, transglutaminase 2 (TG2) induces phosphorylation of FoxO3a at Ser253, inhibiting its nuclear translocation and consequently promoting the synthesis of MMP3 and MMP13 (73). Deacetylases SIRT1 and SIRT3 enhance the nuclear localization and activity of FoxO3a by removing its acetyl modifications (**Table 2**) (67, 71). Dynamic modifications of protein structures, including phosphorylation and acetylation, are key mechanisms regulating FoxO3a activity and chondrocyte fate.

### 5.3 Epigenetics

In tumor research, the epigenetic modifications of FoxO3a have become a significant area of study (74). Certain microRNAs (miRNAs) can target the 3’UTR of FoxO3a, thereby regulating its transcriptional activity (75). Additionally, DNA methylation, histone acetylation, and three-dimensional chromatin remodeling can influence the expression of FoxO3a from various perspectives (76). Similar phenomena have also been observed in the musculoskeletal system. For instance, miR-182 found in synovium-derived exosomes targets the 3’UTR of FoxO3a, inhibiting its expression in human OA synovial stromal cells (**Table 2**) (77). Consequently, the precise regulation of FoxO3a’s expression and localization at the molecular level—through the utilization of signaling pathway cascades, dynamic regulation of PTMs, and specific epigenetic modifications—may represent a highly promising avenue for research.

## 6 Role of FoxO3a in subchondral bone, synovium, and meniscus

The dysfunction of osteoblasts and osteoclasts in the subchondral bone can lead to subchondral bone remodeling, metabolic disorders, angiogenesis, and alterations in innervation. These changes further induce articular cartilage degeneration through mechanisms such as mechanical stress and intercellular communication (78, 79). The differentiation of mesenchymal stem cells into osteoblasts involves a metabolic shift from glycolysis to enhanced mitochondrial respiration. This increase in mitochondrial respiration results in elevated levels of endogenous ROS (80). The upregulation of ROS further activates the phosphorylation of FoxO3a at the Ser294 site, thereby reducing its transcriptional activity and ultimately impairing osteoblast differentiation (81). Moreover, the activation of SIRT1 promotes the deacetylation of FoxO3a, enhancing autophagic flux in mouse osteoblasts and reducing fluoride-induced osteoblasts apoptosis (82). In the subchondral bone of ovariectomy-induced OA (OVX-OA) rats, Sparc secreted by osteoblasts downregulates the AMPK/FoxO3a signaling pathway in chondrocytes, promoting cartilage degeneration through intercellular communication (83).

Synovial fibroblasts play a crucial role in disrupting the joint microenvironment by secreting inflammatory factors and chemokines. Concurrently, the fibrotic process induces irreversible changes in the synovial structure through epithelial-mesenchymal transition (EMT) and collagen deposition (84, 85). Although the role of synovial fibroblasts in OA remains a topic of debate, synovitis is significantly important for OA progression, pain symptoms, and the development of intervention strategies (9, 81). Notably, despite the substantial differences in the pathogenesis of rheumatoid arthritis (RA) and OA, the inactivation of FoxO3a in synovial fibroblasts under the inflammatory conditions of RA exacerbates joint inflammation, providing valuable insights into the pathological mechanisms of arthritis (86, 87).

The meniscus directly influences the mechanical stress and biochemical environment of the joint through dynamic extrusion under load and degenerative tears (88). The expression of FoxO3a is significantly reduced in the menisci of OA patients, aged mice, and destabilization of the medial meniscus (DMM) mice (89). The specific combined knockout of FoxOs in AcanCre inhibits the expression of meniscal autophagy and antioxidant genes, thereby exacerbating meniscal injury and OA progression (**Table 3**).

**Table 3:** Role of FoxO3a in subchondral bone, synovium, and meniscus.

Tissue type	Cell type	Mechanisms	Signaling pathway	Biological functions	Ref.
Subchondral Bone	Human MSCs and osteoblasts	ROS activates FoxO3a phosphorylation	ROS/FoxO3a	Promotes osteoblast differentiation	(81)
	Mouse osteoblasts	SIRT1 promotes deacetylation of FoxO3a	SIRT1/FoxO3a	Increases autophagic flux	(82)
	OVX-OA rat osteoblasts and chondrocytes	Osteoblasts secretion of Sparc downregulates chondrocyte AMPK/FoxO3a	Sparc/AMPK/ FoxO3a	Delays cartilage degeneration	(83)
Synovium	Human RA SFs	Inactivation of FoxO3a in inflammatory environment	TNF/PIK3IP1/FoxO3a and SIRT1/FoxO3a	Inhibits joint inflammation	(86, 87)
Meniscus	OA patients and DMM mice	Inhibition of FoxO3a diminishes autophagy	–	Protects meniscus	(89)

## 7 Applications of targeting FoxO3a in OA diagnosis and treatment

### 7.1 FoxO3a in OA diagnosis

A study integrating bioinformatics analysis and machine learning strategies identified downregulated FoxO3a as a biomarker for OA aging and validated its reliability in the peripheral blood of OA patients (90). Obtaining synovial fluid from the joints of experimental rats is extremely challenging and yields minimal quantities; however, this study found that FoxO3a is downregulated in the joint fluid of OA rats (77). Furthermore, the expression of FoxO3a varies in the joint fluid of OA patients depending on the severity of their condition. Single nucleotide polymorphisms (SNPs) of FoxO3a also contribute to OA diagnosis, with male carriers of the minor allele of FoxO3a quantitative trait loci (QTL) rs4946936 exhibiting a lower risk of developing hip OA (**Table 4**) (91). This study suggests that the expression levels and genetic variations of FoxO3a in peripheral blood and synovial fluid may serve as novel diagnostic biomarkers for OA.

**Table 4:** Applications of FoxO3a in OA diagnosis.

Species	Tissue type	Expression of FoxO3a	Ref.
Human	Peripheral blood	mRNA levels significantly decrease	(90)
Rat/human	Joint fluid	Protein levels show varying degrees of decrease among different OA patients	(77)
Human	Peripheral blood	Male carriers of FoxO3a QTL rs4946936 minor allele have a lower risk of hip OA	(91)

### 7.2 FoxO3a agonists in OA treatment

Currently, several natural or synthetic small molecules have been identified that can directly or indirectly activate FoxO3a in chondrocytes. For instance, NBP and glucosamine activate FoxO3a by inhibiting the PI3K/AKT pathway, which in turn reduces chondrocyte apoptosis and promotes autophagy, demonstrating beneficial effects in both OA rats and human chondrocytes (44, 62). TMF facilitates the nuclear translocation of FoxO3a through the SIRT1/FoxO3a axis, inhibits the expression of ADAMTSs, reduces matrix degradation, and suppresses cartilage hypertrophy (65, 71). USP3 induces the deacetylation of FoxO3a by activating SIRT3, thereby attenuating IL-1β-induced senescence in rat chondrocytes (67). AMPK agonists, such as AICAR and A-769662, upregulate FoxO3a expression by activating the AMPK/FoxO3a signaling pathway, which inhibits oxidative stress and catabolism in chondrocytes, further mitigating cartilage damage in OA (30, 83). Punicalin, a pomegranate extract, exhibits therapeutic effects on IL-1β and TNF-α-induced metabolic disorders in mouse chondrocytes and cartilage by enhancing the transcriptional activity of FoxO3a (32). Additionally, platelet-rich plasma (PRP) has been shown to inhibit apoptosis and promote autophagy by enhancing FoxO3a expression in human OA chondrocytes (**Table 5**) (92).

**Table 5:** Applications of targeting FoxO3a in OA treatment.

Type	Models	Intervention methods	Mechanisms	Functions	Ref.
Agonists	OA rats/human chondrocytes	NBP/Glucosamine	Inhibit PI3K/AKT pathway to activate FoxO3a	Promote ECM synthesis	(44, 62)
	Human OA chondrocytes/OA rats	TMF	Activate SIRT1 to promote FoxO3a deacetylation	Reduce ECM degradation	(65, 71)
	IL-1β-treated rat chondrocytes	USP3	Activate SIRT3 to induce FoxO3a deacetylation	Inhibit cellular senescence	(67)
	Mouse chondrocytes/OVX-OA rat chondrocytes	AICAR/A-769662	Activate AMPK to enhance FoxO3a activity	Inhibit oxidative stress	(30, 83)
	IL-1β or TNF-α-treated mouse chondrocytes	Punicalin	Enhance transcriptional activity of FoxO3a	Inhibit apoptosis	(32)
	Human OA chondrocytes	PRP	Promote FoxO3a expression	Promote autophagy	(92)
Gene Therapy	ATDC5 Cells and human chondrocytes	Lentiviral overexpression of FoxO3a	Induce expression of ACAN	Enhance anabolism	(29, 48)
	DMM rats	Nanotechnology hydrogel delivery of FoxO3a	Enhance secretion of ACAN	Inhibit subchondral bone remodeling	(31)
	ATDC5 cells	siRNA knockdown of FoxO3a	Inhibit expression of COL2A1	Inhibit anabolism	(48)

It is noteworthy that the application of small molecule compounds often requires high bioavailability. For instance, Zhang et al. achieved a high local concentration through intra-articular injection in rats. However, the delivery specificity of FoxO3a agonists presents another challenge. Currently, most systemic administrations of FoxO3a agonists do not utilize specific tools targeting the knee joint, making it unclear what effective concentration reaches the knee joint cartilage (**Table 6**). These studies have laid a foundation for the development of FoxO3a-targeted drugs, but further preclinical and clinical trials are necessary to evaluate their efficacy and safety in OA treatment.

**Table 6:** Limitations of FoxO3a-targeted therapeutic approaches.

Type	Therapeutic approaches	Limitations	Ref.
Agonists	Intra-articular injection	Invasive procedures and non-unique downstream drug targets	(44)
	Gavage/intraperitoneal injection	Off-target risks, low drug availability and non-unique downstream drug targets	(62, 65)
Gene Therapy	Intra-articular injection	Invasive procedures, immunogenicity and ethical controversies	(31)

### 7.3 Gene therapy targeting FoxO3a in OA treatment

As a revolutionary medical technology, gene therapy has demonstrated significant potential in treating both inherited and acquired diseases (93–95). In the context of OA, gene therapy primarily involves the localized delivery of genes to joint tissues to modulate inflammatory responses or promote cartilage repair (96–98).

Studies have demonstrated that lentivirus-mediated overexpression of FoxO3a can induce the expression of ACAN and COL2A1, and reverse IL-1β-induced chondrocyte apoptosis and inflammatory responses (29, 48). Additionally, the delivery of FoxO3a to the knee joint cavity using FoxO3a-NETT@SM effectively ameliorates progressive OA in DMM rats (31). Conversely, the siRNA knockdown of FoxO3a results in increased apoptosis and decreased anabolism in ADTC5 cells (**Table 5**) (48).

Gene therapy faces numerous challenges, particularly the immunogenicity induced by viral vectors and gene editing tools, which can easily trigger host immune responses. This reaction may reduce therapeutic efficacy or even cause toxic side effects (99). Additionally, achieving long-term stable expression of FoxO3a presents a significant technical hurdle that must be addressed in gene therapy. Chen et al. utilized hydrogels to deliver nano-engineered FoxO3a plasmids, leveraging the sustained-release properties of the material to enhance long-term stable expression of FoxO3a. Furthermore, gene editing technology is still mired in ethical controversies (**Table 6**). While gene therapy encounters challenges related to delivery efficiency, targeting specificity, and long-term safety, continuous technological advancements are anticipated to establish it as a crucial direction in future OA treatment.

It is noteworthy that current clinical research on FoxO3a in OA primarily focuses on assessing its expression levels and genetic polymorphisms, with active regulatory strategies for FoxO3a still in the animal experimentation phase. Consequently, more preclinical research data are necessary to support the application of FoxO3a in clinical studies.

## 8 Promising areas for future research

### 8.1 Precise regulatory approaches targeting FoxO3a

As a critical transcription factor, FoxO3a plays a vital role in regulating ECM homeostasis, oxidative stress response, and cell survival in chondrocytes. However, its systemic activation may pose uncontrollable risks. To develop more precise, efficient, and safe regulatory approaches for FoxO3a, the following strategies are expected to be the focus of future research.

The first strategy involves the tissue-specific regulation of FoxO3a. On one hand, tissue-specific delivery systems, such as nanoparticle carriers and ligand-coupling technology, can be developed to precisely deliver FoxO3a activators to OA joints or cartilage, thereby avoiding systemic exposure. On the other hand, cartilage-specific promoters, such as COL2A1, can be utilized to construct gene therapy vectors that achieve specific expression of FoxO3a in chondrocytes (100).

The second strategy is the multi-target combination therapy involving FoxO3a. Combining FoxO3a with upstream and downstream pathway regulators, such as AMPK or PI3K/AKT, can facilitate personalized treatment for OA. For instance, the combination of PI3K/AKT inhibitors MK-2206 and LY294002 in human chondrocytes has been shown to inhibit dexamethasone-induced FoxO3a upregulation and apoptosis (101).

Lastly, the dynamic regulation of FoxO3a activity was discussed. The development of light-controlled and chemically inducible regulatory systems has enabled spatiotemporal control of FoxO3a activity (102). For instance, pH-responsive drug delivery systems can leverage the acidic characteristics of the hypoxic microenvironment in cartilage to modulate the release rhythm of FoxO3a activators.

### 8.2 Downstream target genes of FoxO3a

A comprehensive identification and validation of the downstream target gene network of FoxO3a in chondrocytes is fundamental for understanding and harnessing its functions. In addition to known antioxidant enzymes, apoptosis/autophagy-related proteins, and matrix metabolic enzymes, high-throughput sequencing technologies (e.g., ChIP-seq, RNA-seq) should be employed to systematically explore new target genes that are crucial to OA pathological processes (103). Systematic identification and validation of the downstream target genes of FoxO3a not only elucidates its role in OA but also provides potential targets for the development of innovative therapeutic strategies.

### 8.3 Multi-omics research combined with network analysis of FoxO3a

With the rapid advancement of omics technologies, investigating the mechanisms of FoxO3a in OA through multi-omics research is poised to become a significant focus for future studies. By integrating transcriptomic, proteomic, and metabolomic data, researchers can achieve a more comprehensive understanding of the precise regulatory network of FoxO3a within the specific pathological microenvironment of OA. For instance, RNA-seq technology can be utilized to identify transcriptomic changes associated with FoxO3a expression, and when combined with proteomic analysis, it can elucidate alterations in its downstream signaling pathways (83). Single-cell omics further facilitates the exploration of the expression, functional differences, and intercellular communication of FoxO3a across various cell types. Additionally, network biology approaches, including network pharmacology, assist in constructing the regulatory network of FoxO3a and identifying its interactions with other key molecules, thereby revealing its systemic role in OA.

## 9 Discussion

**Figure 2:**
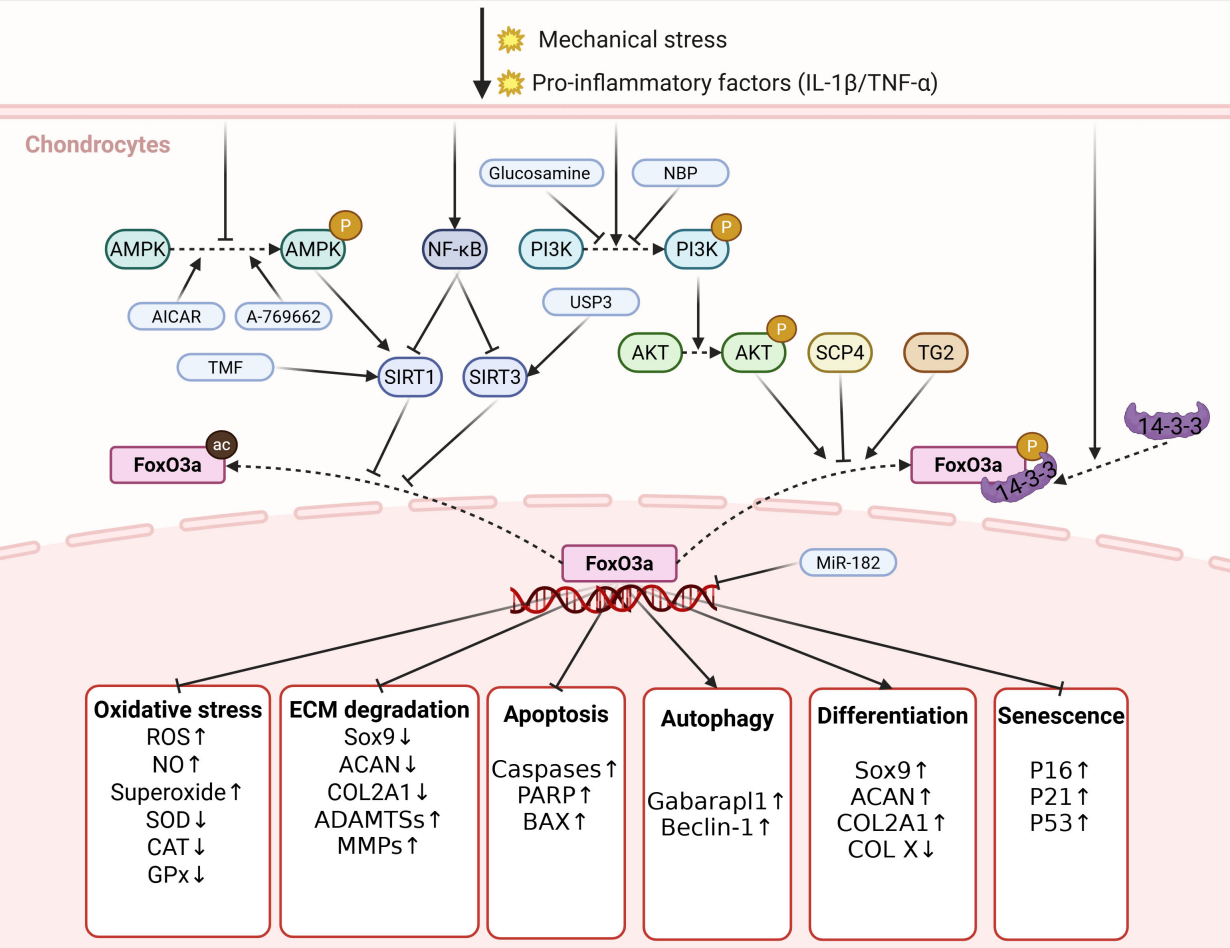
The regulation and biological functions of FoxO3a in chondrocytes. Various stressors, including mechanical stress and pro-inflammatory factors, regulate the PTMs and subcellular localization of FoxO3a in chondrocytes via distinct signaling pathways, which reduces its nuclear activity expression of FoxO3a. The reduced activity of FoxO3a further leads to processes, such as imbalance in redox homeostasis, degradation of ECM and apoptosis of chondrocytes, by regulating the transcription of target genes, ultimately promoting cartilage degeneration and the development of OA. P, phosphorylation; ac, acetylation. Created in https://BioRender.com.

As a crucial transcription factor, FoxO3a plays a significant protective role in the occurrence and progression of OA by regulating various aspects, including redox homeostasis, ECM metabolism, apoptosis, autophagy, and differentiation in chondrocytes. Various stressors, such as mechanical stress and pro-inflammatory factors, regulate the PTMs and subcellular localization of FoxO3a in chondrocytes through different signaling pathways, thereby downregulating its nuclear activity. The decreased activity of FoxO3a further induces processes including chondrocyte apoptosis, imbalance of oxidative-reduction homeostasis, and ECM degradation by regulating the transcription of target genes, ultimately promoting cartilage degeneration and OA development (**Figure 2**).

Unlike Wu et al., who concentrated on the regulatory mechanisms of FoxO3a, and Ma et al., who explored the impact of FoxOs on bone metabolism, this paper primarily focuses on developing therapeutic strategies for FoxO3a and investigating future research methodologies (104, 105). FoxO3a agonists and gene therapy strategies are expected to establish a research foundation for its clinical application. Additionally, a multi-omics integration strategy can systematically screen for downstream target genes and upstream regulatory factors of FoxO3a.

Given the complexity of the pathogenesis of OA and the significant role of FoxO3a in various pathological changes across different tissues, it is essential to recognize that FoxO3a is merely one of the factors contributing to the development and progression of OA (6, 16). A deeper understanding of the role of FoxO3a in OA, particularly in chondrocytes, can enhance our comprehension of the pathological changes associated with OA. However, this understanding must be integrated with the intricate pathophysiological mechanisms underlying OA.

As a classical longevity factor, the dysregulation of FoxO3a expression in relation to aging and OA warrants thorough exploration. Numerous studies have indicated alterations in FoxO3a expression in aging organisms, along with the impact of regulating FoxO3a on delaying the aging process, suggesting a bidirectional relationship between FoxO3a downregulation and aging (15, 16, 31, 66). Considering the pivotal roles of both in the onset and progression of OA, we can hypothesize that a positive feedback regulatory mechanism exists between FoxO3a downregulation and biological aging, which jointly drives the progression of OA (1).

It is noteworthy that FoxO3a should not be regarded solely as a protective factor for OA. Research by Wang et al. indicates that FoxO3a can also promote apoptosis in rabbit chondrocytes and exacerbate oxidative stress (33). After comparing methodological differences with other similar studies, we believe that this phenomenon may arise from species differences or specific experimental conditions. The role of FoxO3a may depend on its microenvironment, and its functions may significantly differ in various states such as chondrocyte homeostasis, senescence, and oxidative stress. In any case, the precise role of FoxO3a in OA requires further investigation.

The modes of administration include local delivery methods, such as intra-articular injection, and systemic delivery methods, including gavage and intraperitoneal injection. While systemic administration of FoxO3a is considered less traumatic than intra-articular injection, it is important to note that systemic delivery carries the risk of off-target effects. Furthermore, the systemic activation of FoxO3a may induce unpredictable physiological changes in various tissues and organs, necessitating rigorous monitoring for potential adverse reactions.

Although the role of FoxO3a varies across different biological conditions and therapeutic contexts, current studies have highlighted its potential as both a diagnostic and therapeutic target for OA. By activating its protective functions, including antioxidant and pro-autophagy mechanisms, it is feasible to effectively delay the progression of OA. This strategy shows promising prospects for application. Future research needs to more precisely dissect the regulatory network and functional specificity of FoxO3a in the pathological microenvironment of OA, elucidate its specific role differences in various disease stages and tissues, and strive to develop methods that can accurately and selectively regulate FoxO3a activity, aiming to maximize its therapeutic potential while minimizing potential adverse effects. A deeper understanding of the biological roles and regulatory mechanisms of FoxO3a in OA will provide important theoretical foundations for the development of new OA prevention and treatment strategies.
